# The strengths of families in supporting mentally-ill family members

**DOI:** 10.4102/curationis.v38i1.1258

**Published:** 2015-04-10

**Authors:** Masego C. Mokgothu, Emmerentia Du Plessis, Magdalena P. Koen

**Affiliations:** 1 School of Nursing Science, North-West University, Potchefstroom Campus, South Africa

## Abstract

**Background:**

Although families caring for a mentally-ill family member may experience challenges, some of these families may display strengths that help them to overcome difficulties and grow even stronger in caring for their family member. In cases where these families are unable to cope, the mentally-ill family member tends to relapse. This indicated the need to explore the strengths of families that cope with caring for mentally-ill family members.

**Objective:**

The purpose of this study was to explore and describe the strengths of families in supporting mentally-ill family members in Potchefstroom in the North-West Province.

**Method:**

A qualitative, explorative, descriptive and contextual design was employed, with purposive sampling and unstructured individual interviews with nine participants. Tesch's eight steps of thematic content analysis were used.

**Results:**

Twelve themes emerged from the data. This involved strengths such as obtaining treatment, utilising external resources, faith, social support, supervision, calming techniques, keeping the mentally-ill family member busy, protecting the mentally-ill family member from negative outside influences, creative communication, praise and acceptance.

**Conclusion:**

Families utilise external strengths as well as internal strengths in supporting their mentally-ill family member. Recommendations for nursing practice, nursing education and for further research could be formulated. Psychiatric nurses should acknowledge families’ strengths and, together with families, build on these strengths, as well as empower families further through psycho-education and support.

## Introduction

Families who take care of a mentally-ill family member might experience immense difficulties (Hughes 2005). These families are in desperate need of support and understanding (Hughes 2005). Lately, the tendency of caring for a mentally-ill family member at home is common because of the high costs of institutional care, poor institutional care and limited availability of institutional care (Sreeja *et al*. 2009).

The main goal of taking care of a mentally-ill family member at home is ‘to enable people with mental health problems to integrate into society and lead normal lives’ (Hsiao & Van Riper 2010). As a consequence of this unexpected shift, the families of mentally-ill family members were given an unexpected caregiving role for which they were both untrained and unprepared (Corsentino *et al*. 2008). As a result of this, families and healthcare providers had to form new partnerships to provide care to mentally-ill family members.

Caring for an individual with a mental illness in the community poses a heavy burden for caregivers (Zauszniewski, Bekhet & Suresky 2009). Caring for a mentally-ill family member takes time, is expensive and is energy consuming (Jiji 2007). De Abreu Ramos-Cerqueira *et al*. (2008) report that families of mentally-ill family members may experience psychological problems and impaired quality of life as they usually have to support their family member alone and without formal training or support. The person with the mental illness can dominate the entire family through control and this can result in fear or helplessness and incapacity on the part of family members (Gravitz 2001).

However, Wynaden (2007) has identified four main reasons why families are committed to caring for their mentally-ill family members: families have an obligation to care; they own their difficulties; they must protect vulnerable members; and they are self-reliant. They are a reliable resource in the sense that they provide food, support, and rehabilitation of a sick family member along with their own needs, care for physical needs, and financial support (Wynaden 2007). They also monitor symptoms, help with management of medication and get treatment on the patient's behalf (Rose, Mallinson & Walton-Moss 2002). These reasons cause families to continue to care for their mentally-ill family members, even when caring is costly and difficult.

Despite the challenges faced by these families, they seem to have resources, knowledge and skills to call upon in difficult times, such as traditions and rituals (Anuradha 2004). They also share and derive strength from other embedded systems, such as extended family and social systems (Njue, Rombo & Ngige 2007). Extended families might include the relatives and other people with a connection to the family, whilst social systems include ‘economic, educational and other resources available to the families within a given culture’ (Njue *et al*. 2007). Families also seem to have sufficient knowledge to define their situations and are able to join up and offer both potential and actual solutions (Anuradha 2004).

Even though these families have strengths and are acknowledged for taking care of their mentally-ill family members, few start out fully prepared – emotionally, physically and financially – to face this situation (O’Grady 2004). By recognising and building on family strengths, families can be empowered. This, according to O’Connell (2006), can be done by helping families to define their situations, give meaning to their situations and adopting a strengths-based approach.

Such families need an understanding of what they are experiencing so that adequate and appropriate support can be offered (Hughes 2005). This, according to Anuradha (2004:389), can be accomplished by understanding family dynamics such as cohesion, flexibility and communication in the family as a system. Family cohesion is defined as ‘the emotional bond that family members feel toward other family members. This is expressed through commitment and spending time together, especially during family events such as weddings, births’, deaths and illnesses (Njue *et al*. 2007).

Family flexibility is the ability to change and adapt to family processes, such as growth, development and aging of family members (Njue *et al*. 2007). It is also expressed by coping with ‘non-normative events’ such as the illness or death of a family member which causes the family to experience stress. Family communication allows for sharing of information and of the feelings, both negative and positive, that family members have for each other (Njue *et al*. 2007). Through adequate communication, family members are able to express their ‘mutual caring and interdependence’ (Njue *et al*. 2007).

### Problem statement

Earlier research findings on family caregiving of mentally-ill family members have indicated that some of these family caregivers lack both an understanding of and the skills related to effective management of mental illness (Seloilwe 2006). Families seem to lack professional support, resources and community support. They need education about mental illness, training in effective caregiving and strategies, as well as the formation of self-help groups in the community (Jiji 2007).

Although most families experience challenges, some ‘are still able to demonstrate individuality, purpose and strength of character’ (Wynaden 2007). They share the ability to overcome the hardship, not only to survive the day-to-day burden associated with caring for a mentally-ill family member, but also to grow into a stronger, more flexible and healthy family (Zauszniewski *et al*. 2009). During the researcher's practice as a psychiatric nurse, it became evident that some of the families of mentally-ill family members are able to cope with taking care of such a patient, whilst others are unable to do so. In cases where the families are unable to cope, the mentally-ill family member tends to relapse and this in turn leads to repeated admissions (Du Plessis, Greeff & Koen 2004). This indicated the need to explore the strengths of families that cope with caring for mentally-ill family members. The families of mentally-ill family members live with and have to assume total responsibility for their ill relatives, yet little is known about their strengths in these circumstances (Seloilwe 2006).

#### Research question

What are the strengths of families in supporting mentally-ill family members?

#### Research objective

The research objective for this study was to explore and describe the strengths of families in supporting mentally-ill family members. Such information might guide the formulation of recommendations which would allow families to support their mentally-ill family members.

## Research design

### Research approach and method

An explorative, descriptive and contextual qualitative research design was used to explore and describe the strengths of families who support mentally-ill family members. Qualitative research is more concerned with ‘the meaning people have constructed, that is, how people make sense of their world and the experiences they have in the world’ (Merriam 2009). Qualitative researchers are interested in understanding how people interpret their experiences, how they construct their world and what meaning they attribute to their experiences (Merriam 2009).

#### Population and sampling

The population studied in this research was family members of mentally-ill patients admitted to a psychiatric hospital in Potchefstroom in the North-West Province of South Africa. Purposive sampling was employed to select potential participants. This means that a researcher selected specific participants, in this case the strengths of families in supporting a mentally-ill family member, who would provide rich data in order to gain insight and discover new meaning (Burns & Grove 2009). Families were recruited via operational managers and at family conferences in a psychiatric unit of a psychiatric hospital rendering services to mentally-ill persons. The sample size was established by data saturation (Burns & Grove 2009).

#### Data collection

The strengths of the families of mentally-ill family members were explored by using unstructured individual interviews. These consisted of an initial open-ended question with one family member of each of the mentally-ill individuals who took part voluntarily in the study. These are interviews that are free-flowing in structure, limited only by the focus of the research (Brink, Van der Walt & Van Rensburg 2006). They are conducted much like a normal conversation, but with a specific purpose in mind (Brink *et al*. 2006). The interview question was: ‘What are your family's strengths in supporting your mentally-ill family member?’ Interviews were conducted at a psychiatric unit, since it is a place that was convenient for the participants and that lent itself to privacy and confidentiality. Participants were asked to give permission for an audio-recorder. The audio-recorded interviews were transcribed for the purpose of data analysis. Communication techniques such as clarifying, summarising and reflection were used to facilitate the discussion.

#### Data treatment

Data captured by the audio-recorder were transcribed and translated from languages such as Setswana, Southern Sotho and Northern Sotho to English and then analysed using codes and coding as well as reflective remarks. This was done by categorising themes, using symbols or abbreviations to classify words in the data. This was done using Tesch's eight steps of thematic content analysis (Creswell 2009). The raw data, as well as a work protocol, were sent to an independent co-coder for analysis and a consensus meeting was held afterwards to identify themes that emerged from the data.

## Ethical considerations

Before conducting the research, the researcher ensured that ethical approval was granted. This research was a sub-study of a larger study that had already been granted ethical approval, entitled *Strengthening of Resilience of Health Caregivers and Risk Groups* (the RISE study) (Koen & Du Plessis 2011). Furthermore, permission to conduct the research was obtained from the North-West Provincial Department of Health and the management of the psychiatric hospital. Upon receiving consent for conducting the research, the researcher approached the operational managers to act as intermediaries. After confirmation from the operational managers, the researcher met the families of those mentally-ill patients who were willing to participate and explained the purpose of the research, the objectives and the expected benefits. Participants were also informed about the right to choose to participate and the right to withdraw from the study at any given time without being penalised. In addition, they were informed about the consent form that had to be signed at the beginning in order to give consent for participation and for the use of an audio-recorder during data collection. Families who were willing to participate were given consent forms to complete prior to collection of data. Information that was obtained from the participants was not shared with people other than the research team.

## Trustworthiness

To ensure trustworthiness of this study, the researcher applied the principles outlined in Guba's model (Krefting 1991). Prolonged engagement was achieved by spending enough time with the participants that they felt comfortable with regard to disclosure of information. The researcher also partnered with the participants so as to explore themes and find answers, to interpret what was heard and respond to (not only absorb) the information. She also actively listened to unexpected disclosure and the expression of deep feelings of participants. Consistency was ensured by exploring the different lived experiences of families supporting mentally-ill family members, which then led to the formulation of guidelines for the support of mentally-ill family members. Finally, neutrality was achieved by involving a co-coder who is experienced in qualitative research by availing her of the raw data and audio-recordings and requesting that she analyse the data independently, after which a consensus meeting took place.

## Results

Data saturation was reached after nine interviews were conducted. The initial planning was to conduct interviews with families but only one member per family was available during data collection to participate on behalf of their families. Initially, the researcher wanted to identify participants via the operational managers of the psychiatric unit, but the researcher was actually assisted by the social workers who were responsible for arranging family conferences. The results of the interviews are represented in Table 1.

**TABLE 1:** Themes identified as family strengths in supporting mentally ill family members.

Theme (arranged according to frequency of occurrence)	Description	Number of reports (frequency of occurrence)
Getting the necessary treatment for the mentally-ill family member	Hospitals, clinics, rehabilitation and counselling	9 reports/100% of participants
Utilising external resources	South African Police Services, churches and traditional healers	8 reports/89% of participants
Spirituality/Faith	Family members invited friends, the mentally-ill family member was invited to the prayer meeting	7 reports/78% of participants
Social support	Support from other family members, support from neighbours	7 reports/78% of participants
Supervising the mentally-ill family member	Keeping the person safe, making sure he/she takes his/her medication, making sure he/she eats enough	5 reports/56% of participants
Finding ways to calm the mentally-ill family member	Talking to him/her politely	4 reports/44% of participants
Explaining the importance of treatment to the mentally-ill family member	The family members explained the need to attend hospital appointments, dangers of smoking dagga, etc.	3 reports/33% of participants
Finding ways to keep the mentally-ill family member busy	Clean the house or prepare food	2 reports/22% of participants
Trying to protect the mentally-ill family member from negative outside influences	For example: ‘Pimps’, friends who use drugs	2 reports/22% of participants
Trying creative ways to communicate with or to understand the mentally-ill family member	Talk to the mentally-ill family member, simple ways to solve problems	2 reports/22% of participants
Giving the mentally-ill family member praise for doing something good/right	Praising the mentally-ill family member	1 report/11% of participants
Accepting the situation	Trusting in God	1 report/11% of participants

### Research findings and literature control

#### Getting the necessary treatment for the mentally-ill family member

All of the participants emphasised the importance of treatment for their mentally-ill family member. Below are some of their statements from the transcripts:

‘We had to take her to the hospital.’ (Interview 6)

‘I then decided to take him to the clinic and they referred him to the hospital.’ (Interview 8)

‘We then took him to the hospital.’ (Interview 4)

‘So I decided to take him to the hospital.’ (Interview 2)

According to the literature, family members and close relatives often assume the role of the caretaker for the mentally-ill family members and take them to treatment, ensuring their compliance with the therapy and their general wellbeing (Carroll 2008).

#### Utilising external resources

Most of the participants reported using external resources as a strength to support their mentally-ill family members. Below are quotations from the transcripts that support these findings:

‘I phoned the police.’ (Interview 8)

Interview 9: ‘At times we would phone the police and they used to take him to the hospital.’ (Interview 9)

‘I once took him to one traditional healer.’ (Interview 4)

‘He was a member of Jehova's Witness church.’ (Interview 5)

Jonsson, Moosa and Jeenah (2009) mention that, according to the *Mental Health Care Act* No. 17 of 2002 (South Africa 2002), members of the South African Police Service must apprehend the mentally-ill person when they have reason to believe – either by personal observation or information obtained from the healthcare professional – that the person is mentally ill and is likely to inflict harm on him-/herself or others.

Adding to this, Sorsdahl, Stein and Flisher (2010) confirm that the families of mentally-ill family members do take their mentally-ill relatives to traditional healers and prefer to be referred to physicians only when the mentally-ill person does not respond to traditional medicine.

#### Spirituality/Faith

Most of the families emphasised their faith in God as an internal strength to support their mentally-ill family member and some families practised their faith/spirituality in the form of prayer. These are some of their statements from the transcripts:

‘Prayed that I wish he could listen to us and quit smoking drugs.’ (Interview 8)

‘We teach them about God.’ (Interview 7)

‘Until such time their cousin explained the situation to the uncle who came with his prayer team and prayed for them.’ (Interview 6)

Interview 4: ‘… [*a*]bsolutely nothing I can do rather than praying for him because it helps a lot.’ (Interview 4)

Scharf (2007) indicates that a sensible religion promotes a positive worldview, helps to make sense of difficult situations, gives purpose and meaning, discourages maladaptive coping (such as the use of drugs and negative activities), enhances social support, promotes other-directedness, helps to release the need for control and also provides and encourages forgiveness, thankfulness and hope.

#### Social support

Some participants reported receiving assistance from their neighbours, for instance, one case where a neighbour brought the mentally-ill family member home because they realised that she wasn't well. Another participant said that the family was helped by an uncle who advised them to approach their situation in a different way in order to help their mentally-ill family member:

‘We were helped by people from the neighbourhood who used to bring her whenever they realised that she wasn't well in her mind.’ (Interview 6)

‘… [*b*]ut now my uncle is my mentor … Uh, he said to me, “approach it like this”.’ (Interview 1)

Interview 3: ‘My mom helped me financially to take him to the traditional healer.’ (Interview 3)

‘He started sleeping at the neighbour's house.’ (Interview 4)

According to Zabow (2008), distressed family members and close relatives are not always fully aware or able to make decisions in crisis situations (acute psychosis, suicide and homicidal behaviours), thus need guidance and advice in these instances.

#### Supervising the mentally-ill family member

Participants stated that they attempt to supervise their mentally-ill family member in order to make sure that he or she is safe, has eaten and has taken his or her medication as prescribed. Below are some of their statements from the transcripts:

‘We are going to make sure that he takes his medication properly by keeping an eye on him.’ (Interview 8)

‘[*I would say*] “Prepare food for yourself”, but he would do that under my supervision.’ (Interview 2)

Similarly, Quinn (2007) found that families stated the need to supervise their mentally-ill family members and that this takes up a great deal of their time, causing them to withdraw from the community.

#### Finding ways to calm the mentally-ill family member

According to the statements below, families confirmed that talking to patients in the appropriate manner helped a lot as they would become more violent and aggressive when spoken to harshly. Others mentioned that calming down the patients when they were uncontrollable helped them to become manageable.

‘We were talking to him in a very appropriate way.’ (Interview 4)

‘I tried to calm him down.’ (Interview 8)

‘I would talk to him politely.’ (Interview 9)

According to Dangdomyouth *et al*. (2008), other strategies employed by families to help mentally-ill family members get better, were to calm them down by using kind words and talking to them gently.

#### Explaining the importance of treatment to the mentally-ill family member

The participants reported that they explain to their mentally-ill family member the importance of going back to the hospital for follow-up, as it was to the advantage of the whole family. Another family reported that they emphasise the dangers of using drugs and encourage their family member to avoid using drugs so as to avoid relapse. Below are quotations from the transcripts that support these findings:

‘We talked to him about the importance of going back to the hospital.’ (Interview 3)

‘I tried to talk to him about the dangers of smoking dagga.’ (Interview 8)

This echoes the findings of Yap, Reavley and Jorm (2012), who found that friends of mentally-ill persons tend to endorse help-seeking behaviour and even assist the mentally-ill person in making appointments to obtain healthcare.

#### Finding ways to keep the mentally-ill family member busy

Families mentioned keeping their mentally-ill family member busy by giving them something to do to keep their mind turned from thinking about doing things they shouldn't. Below are some of their statements from the transcripts:

‘We looked into it that we give her something to do to keep her busy.’ (Interview 7)

‘At times I would tell him to clean the other side of the house while I cleaned the other side.’ (Interview 2)

According to Yap *et al*. (2012), friends endorsed increased physical activity as a way of keeping the mentally-ill family member busy, as it also reflects their exposure to behavioural motivation treatment.

#### Trying to protect the mentally-ill family member from negative outside influences

Participants mentioned that they try to protect the mentally-ill family member from negative outside influences, as is indicated by the following statements:

‘His friend also left school because of drugs. I told him that I don't want to see him with this friend of him [*sic*] any longer.’ (Interview 8)

‘She thought somebody loves her only to find that somebody wants money from her.’ (Interview 7)

Dangdomyouth *et al*. (2008) mentioned that families warned their mentally-ill family members against using strong beverages and alcohol provided by friends and/or community members because it affected their emotional state and triggered psychiatric symptoms.

#### Trying creative ways to communicate with or understand the mentally-ill family member

One participant reported an improvement in the mentally-ill family member's condition after talking to her about her condition. Another participant suggested that the mentally-ill family member should use a compliment or complaints book as a way of expressing her feelings as it seemed as if it was difficult for her to talk about things that were troubling her. See statements below:

‘Ever since we talked about it she is much better.’ (Interview 6)

‘I said to her if you can't speak to me about it, let's find out what's going on … compliments and complaints book … if you don't feel comfortable in approaching me about it, ok, write it in the book … I'll read it.’ (Interview 1)

Tammentie *et al*. (2004) found that the positively-worded statements of the dimensions, such as clear and successful exchange of information between family members in order to clarify meaning and intention, describe the family's health and wellbeing and serve as resources in circumstances where a family has to adapt to new situations.

#### Giving the mentally-ill family member praise for doing something good or right

One participant reported praising their mentally-ill family member as a way of showing appreciation for the good things he did:

‘I even praise him every time after bathing and he would feel happy.’ (Interview 8)

There is no evidence of research concerning praising the mentally-ill member for doing good things. Praise and encouragement are, however, often seen as a positive way of interacting with mentally-ill family members and families are encouraged to do so (National Alliance on Mental Illness 2013).

#### Accepting the situation

Only one participant reported accepting their mentally-ill family member because of their religious belief, as this helped a lot in being able to cope with the situation:

‘I have accepted the situation as a born-again Christian.’ (Interview 6)

According to Lawska *et al*. (2006), mentally-ill family members expect to be noticed, accepted and sympathised with, so a supportive and accepting environment is indispensable for the optimisation of socio-professional therapy and rehabilitation of the ill.

## Discussion

The findings show that families have strengths to support their mentally-ill family members. The most prominent strengths are external in nature, namely the South African Police Services, traditional healers and churches. Although families rely on external resources, they utilise internal strengths as well, namely their faith/spirituality, prayer, praising the mentally ill, keeping the mentally ill busy and calming them down. These strengths help them to accept and cope with caring for their mentally-ill family member.

In addition, families who cope with caring for a mentally-ill family member are involved in several support activities. Firstly, they recognise the mental illness and take the mentally-ill family member to the hospital or clinic. Secondly, they monitor compliance when it comes to medication and follow-up appointments.

It was also evident that the family's reaction toward their mentally-ill family member is similar to Maslow's hierarchy of needs in the sense that it includes physiological needs, need for safety and security, need for love and a feeling of belonging, esteem needs and a need for self-actualisation. It is similar in the sense that the family tries everything in their power to protect their mentally-ill family member. They make sure that they take them to the hospital and/or clinic when they realise that they are behaving strangely, they call the police when they are violent and aggressive or difficult. They try to consult traditional healers, ask friends and relatives to come and pray with them and try to calm them down when aggressive by talking to them politely. In addition, they try to keep them busy by giving them something to do so that they don't roam around aimlessly and praise them when they have done something good so as to show them they love them and that they belong to the family. Families also explain the importance of taking treatment and the dangers of smoking dagga, try creative ways to communicate with or understand their mentally-ill family member and supervise them by making sure that they have eaten and taken their medication. Families also protect them from negative outside influences and show that they accept and love their mentally-ill family member.

Looking more specifically at calming techniques, it seems that it can help families to gain control of their mentally-ill family member since mentally-ill people sometimes become more aggressive and violent when not addressed in the proper way. Praising the mentally-ill family member when he or she does something good or correct can improve the person's wellbeing because the person realises that he or she is accepted and belongs to the family. Finally, when a mentally-ill family member is kept busy all the time, the person may stop roaming around the street and will not engage so easily in substance abuse.

Based on these findings, the recommendations for nursing practice, nursing education and further research could be formulated.

## Limitations of the study

The authors acknowledge that this study was contextual and that the results can't be generalised. However, the results provide valuable insight and recommendations can be considered when supporting families with mentally-ill family members.

## Recommendations

### Nursing practice

Recommendations for nursing practice include guidelines on how to support families in supporting mentally-ill family members. The guidelines follow Maslow's framework, as the findings and conclusions illustrated similarities with this approach. The recommendations below are formulated to fall in line with Maslow's framework.

#### Physiological needs

Psychiatric nurses should us educational programmes to encourage families to continue to maintain the physiological needs of the mentally-ill family members in order to improve their quality of life. Psychiatric nurses should furthermore acknowledge the strengths of families to explain the importance of treatment to the mentally-ill family member and avoidance of habits (such as use of substances) and should collaborate with families to prevent relapse. Psychiatric nursing should furthermore encourage families to continue to be involved in supportive activities, such as to recognise signs and symptoms of mental illness and to take the mentally-ill family member to the hospital or clinic.

#### The need for safety and security

Psychiatric nurses should empower families to maintain the good health of a mentally-ill family member by conducting psycho-education on the nature of the disease, treatment and management, side-effects of prescribed medication, signs and symptoms of relapse, coping skills and appropriate available community resources for dealing with a crisis situation. Psychiatric nurses should furthermore acknowledge families’ strengths with regard to support of the mentally-ill family member, to obtain support from external sources, to supervise the mentally-ill family member, to use calming techniques, to keep him or her busy and to protect him or her. By acknowledging these strengths, psychiatric nurses and families can explore, reflect on and improve on such strengths as ways to support the mentally-ill family member.

#### The need for love and a feeling of belonging

Mentally-ill family members’ needs for love and belonging might be met when families provide support in the form of prayer, involving support from neighbours and traditional healers and by conveying caring through ensuring that the mentally-ill family member obtains treatment and by using calming techniques. These strengths can be acknowledged by the psychiatric nurse and the psychiatric nurse should, in turn, make families aware of these strengths and explore with them the unique ways in which they meet these needs of their mentally-ill family members. Psychiatric nurses should also empower families by encouraging them to join support groups as this can help them to express their own feelings. Listening to others can foster a sense of hope and enhance emotional, physical and psychological wellbeing. In addition, talking to people with similar problems promotes a sense of belonging.

#### Esteem needs

Psychiatric nurses should empower families by helping them to acknowledge their strengths in meeting the esteem needs of mentally-ill family members and to strengthen their communication skills, such as understanding and praise, conflict management skills and maintaining a respectful attitude. This can be done by means of psycho-education and case management.

#### Self-actualisation

From the findings and conclusions it is evident that families do give attention to the self-actualisation needs of mentally-ill family members by praying with them and by exercising their faith and cultural practices. Psychiatric nurses should explore these strengths with families and, if acceptable to the family and mentally-ill family member, encourage prayer, faith and acceptance. Families can be advised to engage in prayer meetings and church activities as this might promote self-actualisation and strengthen the family's ability to support their mentally-ill family member.

In addition, psychiatric nurses should collaborate with the South African Police Force with regard to involuntary hospitalisation of a mentally-ill family member. This will provide families with further support in terms of managing aggressive and violent family members who are mentally ill. Further support to families include that psychiatric nurses should help them by means of training sessions that teach families about sharing tasks and household chores, so that they are able to relieve one another when caring for a mentally ill family member.

### Nursing education

Recommendations for nursing education include that psychiatric nurses should receive training on the strengths of family members in supporting their mentally-ill family members. This will contribute to relevant and appropriate care in collaboration with families and will also lead to improved quality of care and prevention of recurrent re-admissions.

## Conclusion

Based on the research findings of the study it is clear that there is a need for further research on the strengths of families who support mentally-ill family members. Such research should focus on the following: the utilisation of families’ internal strengths to equip them with the positive healthy behaviour required to support the mentally-ill family member; effective health education for families on the diagnosis of the mentally-ill family member; the cause of mental illness and its impact on the daily functioning of a mentally-ill family member; available support groups in the community; effective health education; communication skills between members of the family; and the management of aggressive or violent mentally-ill family members.
